# On the Fractality of Complex Networks: Covering Problem, Algorithms and Ahlfors Regularity

**DOI:** 10.1038/srep41385

**Published:** 2017-01-27

**Authors:** Lihong Wang, Qin Wang, Lifeng Xi, Jin Chen, Songjing Wang, Liulu Bao, Zhouyu Yu, Luming Zhao

**Affiliations:** 1 Faculty of Mechanical Engineering and Mechanics, Ningbo University, Ningbo 315211, P. R. China; 2 Department of Mathematics, Ningbo University, Ningbo 315211, P. R. China; 3 Department of Software Engineering, Zhejiang Wanli University, Ningbo 315100, P. R. China; 4 College of Science, Huazhong Agricultural University, 430070, Wuhan, P. R. China; 5 Faculty of Mechanical Engineering and Mechanics, Ningbo University, 315211, P. R. China

## Abstract

In this paper, we revisit the fractality of complex network by investigating three dimensions with respect to minimum box-covering, minimum ball-covering and average volume of balls. The first two dimensions are calculated through the minimum box-covering problem and minimum ball-covering problem. For minimum ball-covering problem, we prove its NP-completeness and propose several heuristic algorithms on its feasible solution, and we also compare the performance of these algorithms. For the third dimension, we introduce the random ball-volume algorithm. We introduce the notion of Ahlfors regularity of networks and prove that above three dimensions are the same if networks are Ahlfors regular. We also provide a class of networks satisfying Ahlfors regularity.

Complex networks arise from natural and social phenomena such as the Internet, the protein interactions, the collaborations in research, and the social relationships. Readers are referred to Watts-Strogatz’s^1^ small-world network model and Barabási-Albert’s^2^ scale-free network model, and Newman’s review^3^ and book^4^, etc.

In this paper, we revisit the fractality of complex network by investigating three dimensions *d*_*B*_^5^, *d*_*ball*_^6^ and *d*_*f*_^7^ with respect to minimum box-covering, minimum ball-covering and average volume of balls. The compact box burning algorithm (**CBB**)^8^,^9^ and random ball-covering algorithm^6^ are proposed to calculate *d*_*B*_ and *d*_*ball*_ respectively. However the minimum box-covering problem and minimum ball-covering problem are NP-complete, which are proved rigorously in Theorem 1 and Proposition 2 respectively. The NP-completeness implies that the CBB algorithm and the random ball-covering algorithm do not have high performance, then we suggest some algorithms to improve the random ball-covering algorithm. For the third dimension *d*_*f*_, we obtain an efficient algorithm: the random ball-volume algorithm. When do the three dimensions coincide? To answer this question, we introduce the notion of Ahlfors regularity of networks and prove that *d*_*B*_ = *d*_*ball*_ = *d*_*f*_ (Theorem 2) if networks are Ahlfors regular. Then for Ahlfors regular networks, the random ball-volume algorithm is efficient to obtain the above three fractal dimensions.

## Fractal dimensions and covering problems

Song, Havlin and Makse^5^ reveal that many real networks have self-similarity and fractality, and Gallos, Song, Havlin and Makse give a review of fractality of complex networks^10^. The algorithms to numerically calculate the fractal dimension of complex networks have been proposed: For example, the CBB algorithm^8^,^9^ is applied to calculate the fractal dimension of complex networks through the *minimum box-covering*; Kim, Goh, Kahng and Kim^11^ improve the CBB algorithm to investigate the fractal scaling property in scale-free networks; Zhou, Jing and Sornette^12^ propose the edge-covering box algorithm; Gao, Hu and Di^6^ give the *minimum ball-covering* approach to calculate the fractal dimension of complex networks.

Recall some notation. Considering a network as a graph *G* = (*V, E*) equipped with geodesic distance d, we let an *l*-**box** *A* denote a subset of *V* such that the geodesic distance of any two points in the subset is less than *l*, an *l*-**ball** centered at *x*_0_ the subset 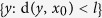. Let *N*_*l*_ be the smallest number of *l*-boxes needed to cover *V*, and *B*_*l*_ the smallest number of *l*-balls needed to cover *V*. Suppose that


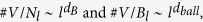


where *d*_*B*_ is the fractal dimension defined by Song, Havlin and Makse^5^, and *d*_*ball*_ is defined by Gao, Hu and Di^6^.

For **box**-**covering**, Song, Gallos, Havlin and Makse^9^ point out that the minimum *l*-box-covering problem is NP-complete for any *l* ≥ 2. On the other hand, for **ball**-**covering**, which is *far from box-covering* in graph theory, we have

**Theorem 1**. *The minimum l-ball covering problem is NP-complete for any l* ≥ 2.

## Ball-covering algorithms

Due to the NP-completeness, for finding the feasible solution of minimum ball-covering problem, we can apply the usual *random ball-covering algorithm* (**RBC**)^6^: when *l* is fixed, in each time *t*, we randomly choose one node *x*_*t*_ in the vertex set *V*_*t*−1_ remained in time (*t* − 1), and obtain *V*_*t*_ by cutting all nodes in 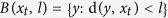.

In the RBC algorithm we give a random sorting for nodes in *V*_*t*−1_ and take the first node. Moreover, given some function 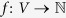, we can sort these nodes according to the values of function *f*.

Given a function 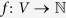, suppose we sort nodes according to values of *f* in nondecreasing order: If *f* is the degree function, we can obtain *degree-order ball-covering algorithm* (**DOBC**); If 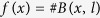 and, we obtain *volume-order ball-covering algorithm* (**VOBC**).

For a function 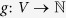, assume we sort nodes according to values of *g* in nonincreasing order, we propose the following greedy algorithm:

1. Assume that 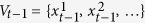 such that 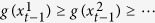.
2. Set 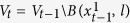 and the sorting of nodes in *V*_*t*_ inherits from *V*_0_ = *V*.

When 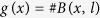, we obtain the *volume-greedy ball-covering algorithm* (**VGBC**). Let *g(x*) = deg(*x*), we have the *degree-greedy ball-covering algorithm* (**DGBC**).

In the point of view on fractal geometry, the box dimension is independent of the geometric shapes of covering, such as ball or box. It is easy to check that *B*_*l*_ ≤ *N*_*l*_ ≤ *B*_*l*/2_, hence 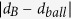 ≤ 
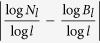
 ≤ 
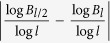
 ≈ 
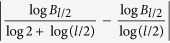
 ≈ 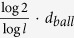. By the above estimate, when the diameter of network is large enough to insure that *l* can be taken large enough, we have

**Proposition 1**. *The fractal dimensions d*_*B*_ *and d*_*ball*_ *w.r.t. the box covering and ball covering respectively are the same*.

However, for real networks with small-world effect, we can not take *l* large enough, and the upper bound 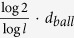 of error is not small enough. On the other hand, we only find the feasible solutions of minimum covering problems due to their NP-completeness. See the following example.

**Figure 1:**
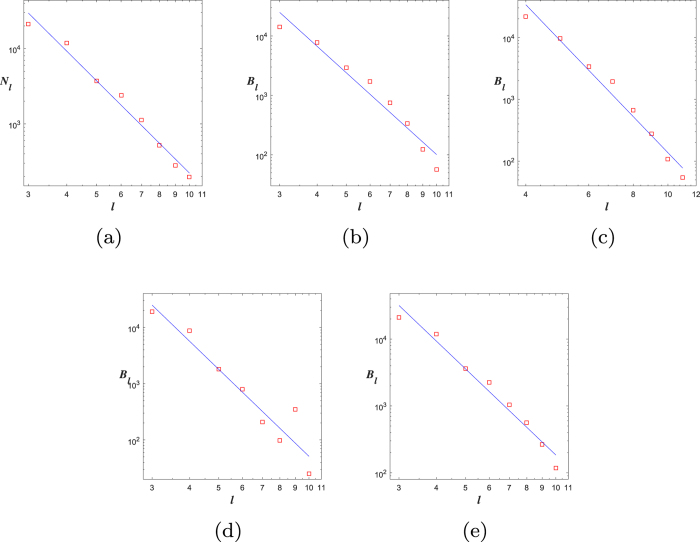
Slopes exist w.r.t. 5 algorithms for the WWW network: (**a**) RBC, (**b**) DGBC, (**c**) DOBC, (**d**) VGBC, (**e**) VOBC.

**Example 1**. *Through above* 5 *algorithms* (Fig. 1), *we calculate d*_*ball*_ *for the WWW network* (Table 1).

*In* Table 1, *the value of the RBC algorithm is exactly the value d*_*ball*_ = 4.2 *by Gao, Hu and D*i^6^. *Note that Song, Havlin, and Makse*^5^ *obtain that d*_*B*_ = 4.1.

**Figure 2:**
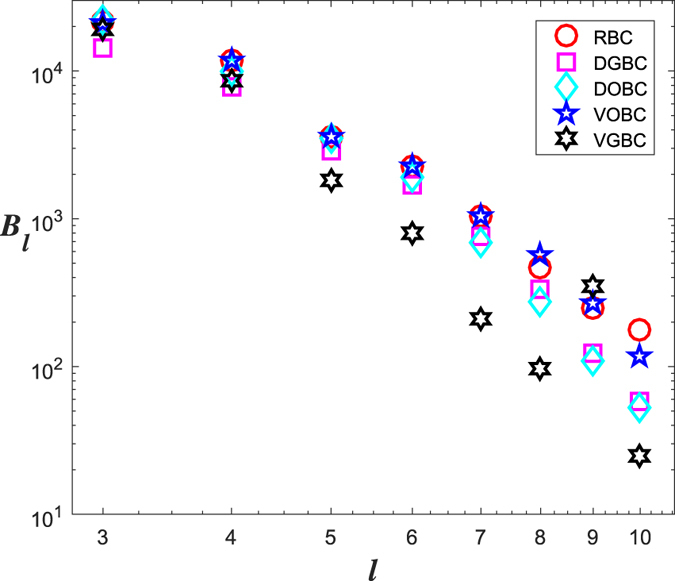
Comparison of 5 algorithms for the WWW network.

*For the WWW network, we also compare the above* 5 *algorithms* (Fig. 2). *It seems that the VGBC algorithm is the best and the performance of the RBC is the worst and close to the VOBC*.

## Random ball-volume algorithm

Based on Shanker’s work^13^, Guo and Cai^7^ investigate the power law between the average volume of balls and the their radii. Given a network, let *p(l*) be the average cardinality of nodes in a ball with radius *l*, suppose that


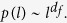


We call *d*_*f*_ the volume dimension. Please also see generalized volume dimension^14^ by Wei *et al*.

We will discuss the volume dimension *d*_*f*_ related to average ball-volume and propose the random ball-volume algorithm for networks. Compared with the minimum box-covering algorithm and the minimum ball-covering algorithm, we have the following algorithm to calculate the average volume of ball with size *l* approximately.

*Random ball-volume algorithm* (**RBV**) (for fixed size *l*):

1. Randomly take a node *x* in the network.
2. Calculate the volume *ν(B(x, l*)).
3. Repeat the steps 1–2 and obtain average volume of random *l*-balls.

**Figure 3:**
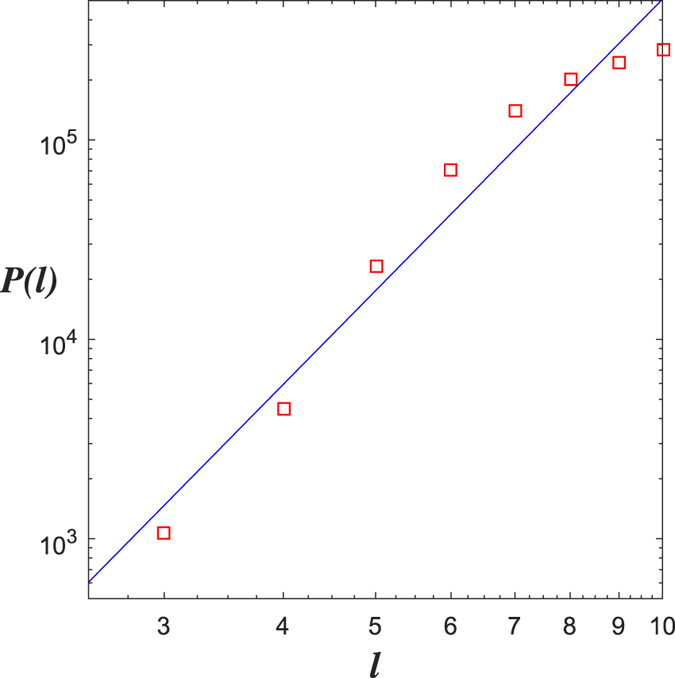
RBV for the WWW network.

For the WWW network, using the RBV algorithm we obtain *d*_*f*_ = 5.833 (Fig. 3).

## Ahlfors regularity of networks

Fractal geometry and fractal network have deep connection. We can generate complex network models from self-similar fractals. For example Andrade *et al*.^15^ and Zhou *et al*.^16^ discuss Apollonian networks generated from Apollonian fractal, Zhang *et al*.^17^,^18^,^19^ construct evolving networks modeled from Sierpinski gasket by taking the line segments as nodes. Besides Zhang *et al*.^20^ construct the networks produced from Vicsek fractals, Liu *et al*.^21^ and Chen *et al*.^22^ explore some Koch networks related to Koch curves, Song *et al*.^23^ study complex networks modeled on Platonic solids, Chen *et al*.^24^ investigate networks generated by Sierpinski tetrahedron.

In this paper, we try to find out the farther connection between the fractal networks and fractal geometry. Recall some classical result on fractal dimension. We find out that many dimension results have measure versions. Suppose *μ* is a Borel (finite) measure supported on a compact subset *E*, denoted by spt 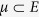. For any 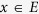, let the lower local dimension of *μ* at point *x* be defined by 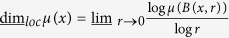. A classical result^25^,^26^ on Hausdorff dimension dim_*H*_ (·) is


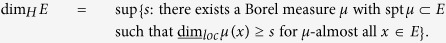


That means for Hausdorff dimension, we have the corresponding measure version. When replacing 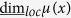 by 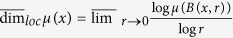, we obtain packing dimension dim_*P*_ (·)^25^,^26^. We always have 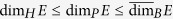, where 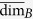 is upper box dimension. A reasonable case is 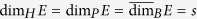 and there is a suitable measure *μ* such that 
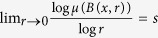
, or we can pose the **Ahlfors regularity** assumption on the measure


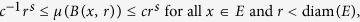


where *c* is an independent constant.

We give a *natural measure* on a graph *G* = (*V, E*). For 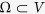, we let *ν*(Ω) be the cardinality of Ω, which is called the *volume* of Ω. We say that {*G*_*t*_}_*t*_ is a family of growing networks, i.e., 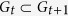, which means the node set of *G*_*t*+1_ contains node set of *G*_*t*_, and neighbors of *G*_*t*_ are still neighbors of *G*_*t*+1_. When {*G*_*t*_}_*t*_ is growing, we let *ν*_*t*_(Ω) denote cardinality of 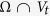, where *V*_*t*_ is the node set of *G*_*t*_.

**Remark 1**. *When taking* 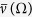 *as the sum of degrees of nodes in* Ω, *Wei et al.*^14^ *obtain the generalized volume dimension*.

**Definition 1**. *Given s* > 0, if 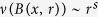, *we call the network an Ahlfors s-regular network. When* {*G*_*t*_}_*t*_ *is growing, we call* {*G*_*t*_}_*t*_ *Ahlfors s-regular networks, if there is an independent constant c such that for all* 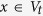, *r* < *diam(V*_*t*_) *and t*,


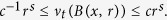


When the diameter of network is large enough, we have

**Theorem 2**. *d*_*f*_ = *d*_*ball*_ = *d*_*B*_ = *s if the network or growing networks are Ahlfors s-regular*.

When the networks are regular, we can use RBV algorithm to obtain their fractal dimensions efficiently.

## Ahlfors regular trees

**Figure 4:**
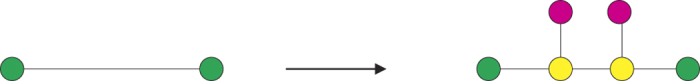
Rule 1.

**Figure 5:**
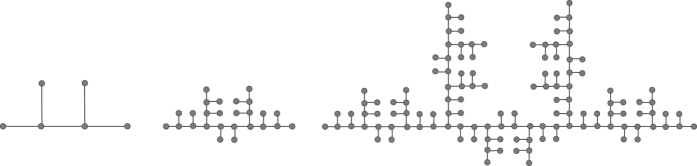
*G*_1_, *G*_2_, *G*_3_ of growing trees w.r.t. rule 1.

**Figure 6:**
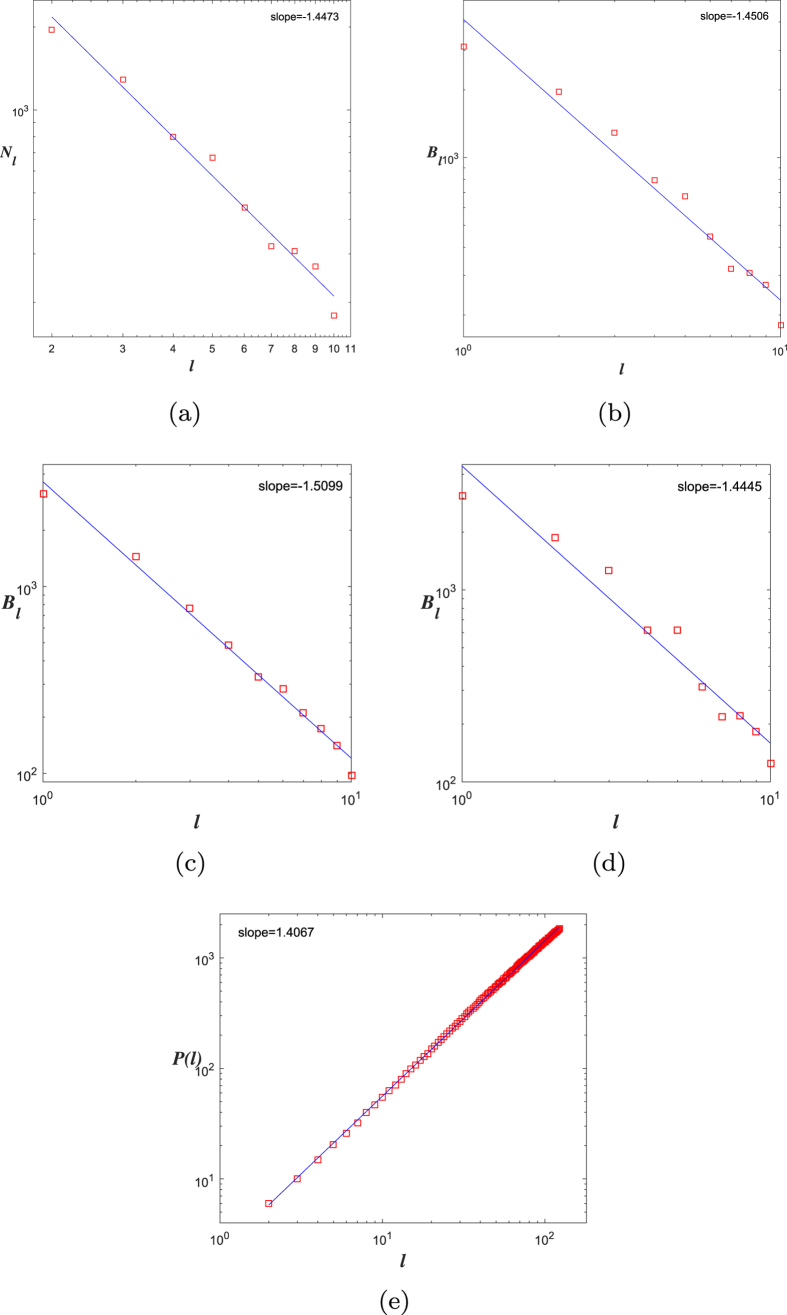
Fractal dimensions of *G*_5_: (**a**) CBB, (**b**) RBC, (**c**) DGBC, (**d**) DOBC, (**e**) RBV.

Now, we obtain a rule (rule 1) of generating Ahlfors *s*-regular networks and growing trees in Figs 4 and 5. We have 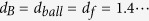 in Fig. 6. By embedding the self-similar tree into the self-similar fractal in 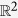, we find that the volume of ball in the tree is comparable with the (self-similar) measure of ball in plane, then we can obtain

**Theorem 3**. *The growing self-similar trees defined above are Ahlfors s-regular with s* = log 5/log 3. *Therefore, we have*


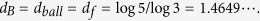


**Figure 7:**
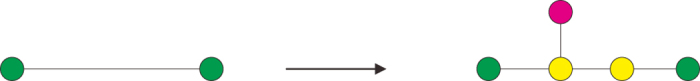
Rule 2.

**Figure 8:**
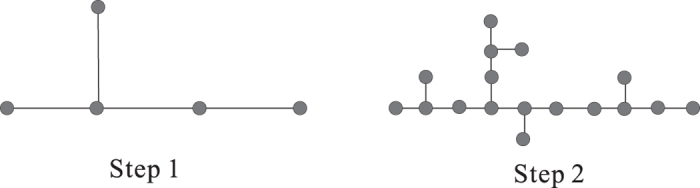
*G*_1_, *G*_2_ of growing trees w.r.t. rule 2.

We also have rule 2 and growing trees in Figs 7 and 8. For this self-similar tree with respect to rule 2, we have 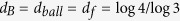.

**Figure 9:**
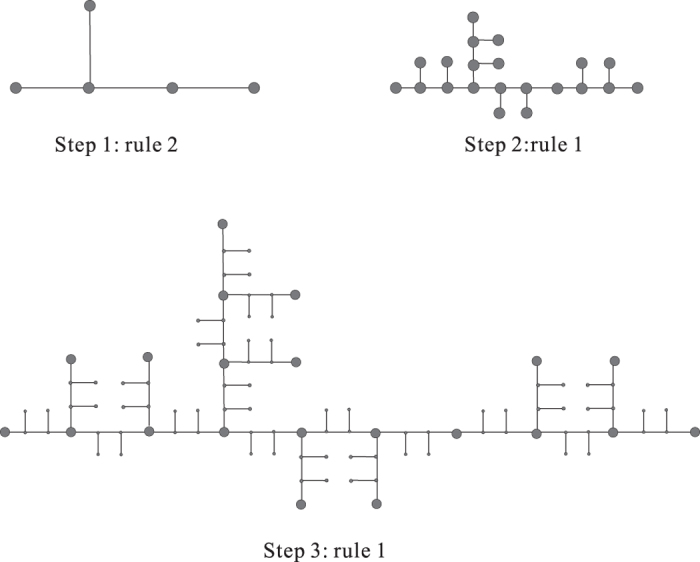
The first three steps according to an infinite sequence 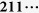.

Fix an infinite sequence 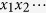 of 1 and 2 such that 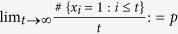 exists. We can construct a family of growing networks as follows by induction: for time *t*, we take rule 1 if *x*_*t*_ = 1, else take rule 2. For example, if the sequence is 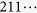, we obtain our growing networks *G*_1_, *G*_2_, *G*_3_ as in Fig. 9. This is a family of deterministic growing networks.

Then we can generate a **Moran tree** with mixed rules. For this Moran tree without self-similarity, we have 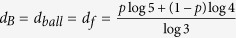. We also obtain **random growing networks**, for each time *t*, we can choose rule 1 in probability *p* and rule 2 in probability 1 − *p*.

The rest of paper is organized as follows. Section 2 is devoted to the rigourous proofs on the NP-completeness of minimum ball-covering problem (Theorem 2) and minimum box-covering problem (Proposition 2). Section 3 is the preliminary on the Ahlfors regularity of fractal geometry, including covering inequality and self-similar fractal. In this section, we also recall the fact that the open set condition of self-similar fractal implies the Ahlfors regularity of fractal measure. Replacing the fractal measures by the cardinalities of subsets of networks, we obtain the Ahlfors regularity of networks. In Section 4, we prove Theorem 2 by using covering inequality shown in Section 2, and obtain Ahlfors regularity of a class of self-similar network (Theorem 3) by constructing bilipschitz mappings from a self-similar fractal, satisfying the open set condition, to self-similar networks, and estimating the cardinalities of balls of graph from the Ahlfors regularity of the fractal measure.

## NP-completeness of minimum covering problems

Recall some notation of computer science. For an alphabet 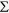, let 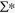 be the set of finite strings of elements of 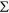, and 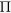 the class of functions from 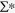 into 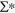 defined by one-tape Turing machine which operate in polynomial time.

**Definition 2**. *Let L and M be languages. Then* 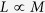 (*L is reduced to M) if there is a function* 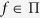 *such that* 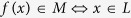. *We say that some language* 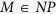 *is **NP**-**complete**, if* 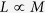 *for all* 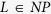.

The concept of NP-completeness was introduced in 1971 by Cook^27^. In Cook’s theorem, he proved that the **Boolean satisfiability problem** is NP-complete.

In 1972, Karp^28^ proved that several other problems were also NP-complete. For example, we give the following two in Karp’s 21 NP-complete problems.

1. **Clique covering problem**
2. Input: graph *G* = (*V, E*), positive integer *k*
3. Property: *V* is the union of *k* or fewer cliques, where a *clique* is a subset of vertices of *G* such that its induced subgraph is complete.
4. **Set covering problem**
5. Input: universe *U* and a family *S* of subsets of *U*, positive integer *k*
6. Property: there is a set covering of size *k* or less, where a *set covering* is a subfamily 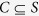 of sets whose union is *U*.
7. In 1992, Kann^29^ proved that the set covering problem, which is NP-complete, can be reduced to the following dominating set problem (hence it’s also NP-complete).
8. **Dominating set problem**
9. Input: graph *G* = (*V, E*), positive integer *k*
10. Property: there is a dominating set of *k* or fewer nodes, where a *dominating set* is a subset *D* of *V* such that every vertex not in *D* is adjacent to at least one member of *D*.
11. In this section, we will show the following two problems are NP-completes.
12. *l*-**ball**-**covering problem**
13. Input: graph *G* = (*V, E*), positive integer *k*
14. Property: *V* is the union of *k* or fewer *l*-balls.
15. *l*-**box**-**covering problem**

Input: graph *G* = (*V, E*), positive integer *k*

Property: *V* is the union of *k* or fewer *l*-boxes.

### Proof of Theorem 1

If *l* = 2, then *l*-ball-covering problem is exactly the dominating set problem, which is NP-complete.

**Figure 10:**
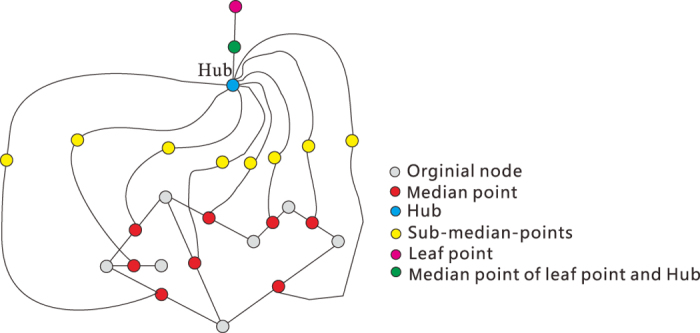
The reduction process for *l* = 3.

If *l* = 3, given a undirected graph *G* = (*V, E*) as in Fig. 10, we construct a new graph 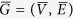 in polynomial time w.r.t. the size of *G*.

1. For any 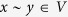, we insert a median point *z* (in red) in the edge 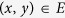 with degree 2 in 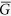, i.e., in 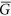 we have *x* ~ *z, z* ~ *y* and *x, y* are not neighbors in 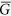.
2. We add a Hub (in blue) to connect all median points.
3. Insert sub-median-point (in yellow) for every edge between one median point (in Step I) and Hub.
4. We construct a leaf node (in pink) and the median point (in green) between leaf node and the Hub.

We have the following

**Claim 1**. *There is a dominating set of k or fewer nodes in G if and only if* 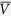 *is the union of (k* + 1) *or fewer* 3-*balls*.

To verify this claim, we notice the following facts.

- For any nodes 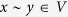, in 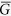 their geodesic distance 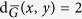.
- The subset of all nodes not in *V* is a 3-ball centered at the Hub.
- The geodesic distance between the pink node and any node in *V* is 5, that means any 3-ball can not contain the pink node and any node of *V* simultaneously.
- For any 3-ball *D* with 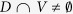, we can find a node *u* in 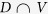 such that

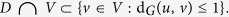


Suppose 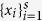 is the minimum dominating set of *G* and there is a minimum 3-ball covering 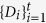 of 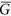. We only need to show that


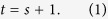


In fact, we have 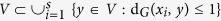. It follows from the fact (a) that


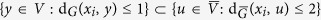


for any *i* ≤ *s*. Applying the fact (b), we see that there exists a 3-ball covering with (*s* + 1) balls. Hence


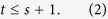


On the other hand, considering the minimum 3-ball covering 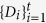, by fact (d), we obtain a dominating set 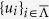 of *G*, where 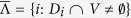. Therefore, 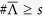. Since the pink point must belong to some ball 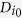, by fact (c), we have 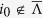. Therefore we have


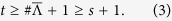


Then (1) follows from (2) and (3). Then Theorem 1 is proved for *l* = 3.

**Figure 11:**
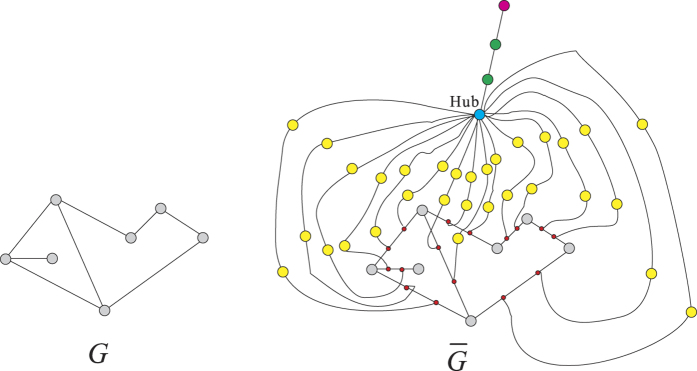
The reduction process for *l* = 4.

For *l* ≥ 4, we have the similar construction during reduction. In fact, we insert (*l* − 2) median points into each edge of *G*, add a Hub to connect all median points, insert (*l* − 2) sub-median-point for every edge between one median point and Hub. Finally, we construct the leaf node and connect it to the Hub, insert (*l* − 2) the median point between leaf node and the Hub. See Fig. 11 for *l* = 4.

**Remark 2**. *To prove one problem is NP-complete, we always find a reduction from a known NP-complete problem to our problem. On the other hand, we can always construct a reduction from our (NP) problem to a known NP-complete problem due to the definition of NP-completeness*.

We give a proof of the following fact which is pointed out by Song, Gallos, Havlin and Makse^9^.

**Proposition 2**. *For any fixed size l, the l-box-covering problem is NP-complete*.

*Proof*. If *l* = 2, *l*-box-covering problem is exactly the clique covering problem, which is NP-complete.

**Figure 12:**
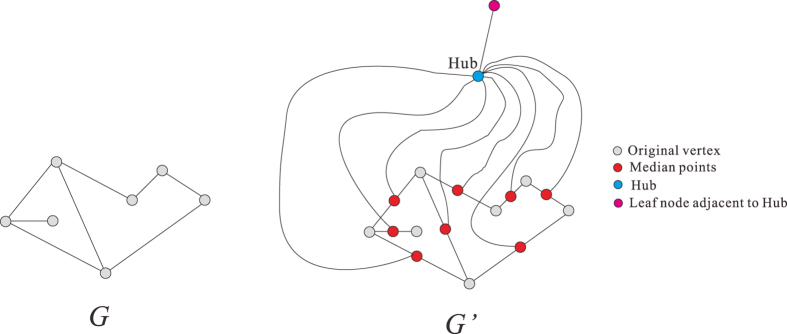
The reduction process for *l* = 3.

If *l* = 3, given a undirected graph *G* = (*V, E*), as in Fig. 12, we construct a new graph *G*′ = (*V*′, *E*′) in polynomial time with respect to the size of *G*.

1. For any 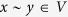, we insert a median point *z* (in red) in the edge (*x, y*) with degree 2, i.e., *x* ~ *z, z* ~ *y* and *x, y* are not neighbors in *G*′.
2. We add a Hub (in blue) to connect all median points.
3. We construct a leaf node (in pink) adjacent to the Hub.

We have the following

**Claim 2**. *V is the union of k or fewer cliques if and only if V*′ *is the union of (k* + 1) *or fewer* 3-*boxes*.

To verify this claim, we notice the following facts.

- For any nodes 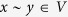, in *G*′ their geodesic distance 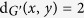.
- The subset of nodes not in *V* is a 3-box.
- The geodesic distance between leaf node (in pink) and any node in *V* is 3.

Suppose 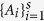 is a family of cliques of *G* such that *s* is minimal one. Suppose there is a minimum 3-box covering 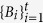 of *G*′. We only need to show that


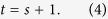


In fact, we have 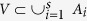. It follows from the fact (i) that *A*_*i*_ is a 3-box in *G*′ for any *i* ≤ *s*. Applying the fact (ii), we see that there exists a 3-box covering with (*s* + 1) boxes. Hence


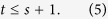


On the other hand, it follows from fact (i) that 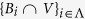 is a family of cliques in *G* where 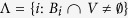. Therefore, 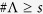. We also notice that if the pink point belongs to some 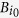, by fact (iii), we have 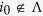. Therefore we have


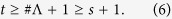


Then (4) follows from (5) and (6).

**Figure 13:**
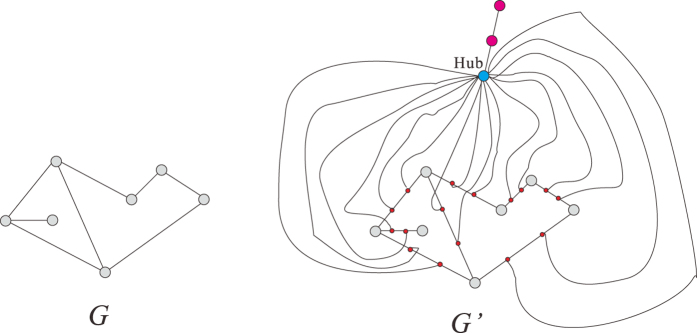
The reduction process for *l* = 4.

For *l* ≥ 4, we have the similar construction during reduction. See Fig. 13 for *l* = 4.□

## Covering inequality, self-similar fractal and Moran fractal

### Covering and packing on metric space

Given a compact metric space (*X*, d), let a *δ*-ball centered at *x*_0_ be an open ball 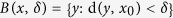, and a *δ*-cube a cube of Euclidean space with side length *δ*, a *δ*-box *B* is a subspace of *X* such that its diameter less than *δ*, i.e., d(*x, y*) < *δ* for all *x*, 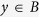. Denote


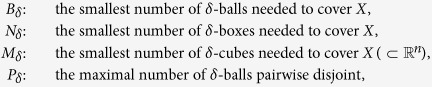


We recall an elementary inequality^26^ which is important in this paper. We give the proof for the self-containedness of this paper.

**Lemma 1**. 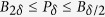.

*Proof*. Suppose 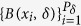 is a packing family of *δ*-balls, we conclude that 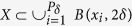. Otherwise, suppose 
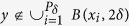
, for any 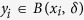, we have d(*y, y*_*i*_) ≥ d(*y, x*_*i*_) − d(*y*_*i*_, *x*_*i*_) ≥ 2*δ* − *δ* = *δ*. That means 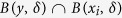 is empty for any *i*. Now, we obtain a new packing family of 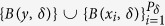, which is a contradiction. Therefore, we have 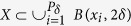, and thus we have *P*_*δ*_ ≥ *B*_2*δ*_.

Assume 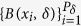 is a packing family of *δ*-balls, then d(*x*_*i*_, *x*_*j*_) ≥ *δ* for all 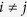. Notice that on Euclidean space, we have d(*x*_*i*_, *x*_*j*_) ≥ 2*δ* for all 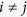. Suppose there is a minimum covering of *δ*/2-balls 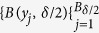. Now, every *δ*/2-ball contains at most one points in 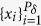 since the diameter of a *δ*/2-ball is less than *δ*and d(*x*_*i*_, *x*_*j*_) ≥ *δ* for all 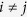. On the other hand, every *x*_*i*_ must be contained in some *δ*/2-ball. Therefore, we obtain *P*_*δ*_ ≤ *B*_*δ*/2_.□

We also have


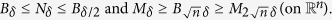


By the above inequalities, the classical result^25^,^26^ on box dimension is that


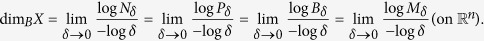


In fact, in the above formula, we take upper box dimension 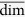 or lower box dimension 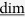 when the limit does not exist.

### Self-similar set on Euclidean space

Let 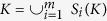 be a self-similar set^30^ on a Euclidean space 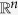, where *S*_*i*_ is a similarity with ratio *r*_*i*_, i.e., 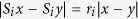 for all *x*, 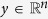. In fact, 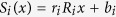 where 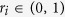, 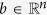 and *R*_*i*_ is orthogonal. That means any similarity is the compositions of homothety, translation and orthogonal transformation.

We say that the open set condition (**OSC**) holds if there exists a non-empty open set *V* such that


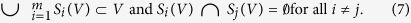


Let 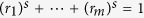 and the probability vector 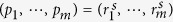. According to ref. 30, there is a unique Borel measure *μ* (self-similar measure) satisfying 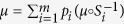. When the OSC holds, Hutchinson^30^ obtained that dim_*H*_ *K* = dim_*B*_ *K* = *s*, and there is a constant *C* ≥ 1 such that for all 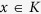 and *r* ≤ |*K*| (the diameter of *K*),


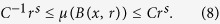


A compact set *E* is said to be Ahlfors *s*-regular^26^, if there is a Borel measure *μ* supported on *E* satisfying (8). That means the self-similar set satisfying the OSC is Ahlfors regular.

### Self-similar fractals

**Figure 14:**
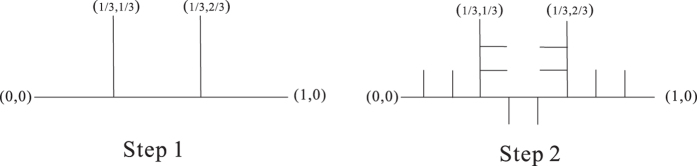
The first two steps of self-similar fractal (model 1).

**Figure 15:**
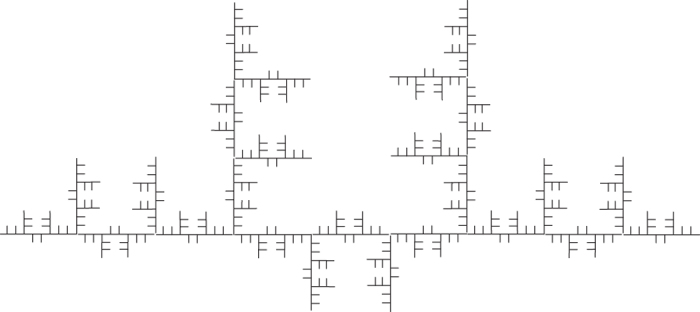
Step 4 of self-similar fractal (model 1).

We introduce a special self-similar fractal on 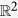 (Figs 14 and 15). Let


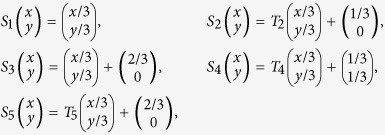


where orthogonal. matrixes 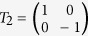, 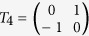, 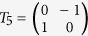.

**Figure 16:**
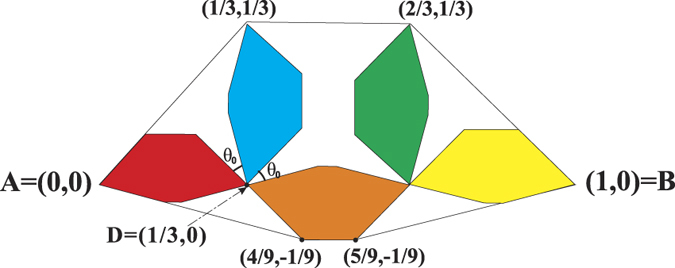
OSC holds.

Let *V* be the interior of polygon with vertexes (0, 0), (1/3, 1/3), (2/3, 1/3), (1, 0), (4/9, −1/9) and (5/9, −1/9). Then (7) holds for *m* = 5 (Fig. 16).

**Figure 17:**
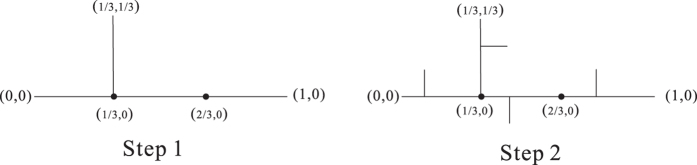
The first two steps of self-similar fractal of model 2.

**Figure 18:**
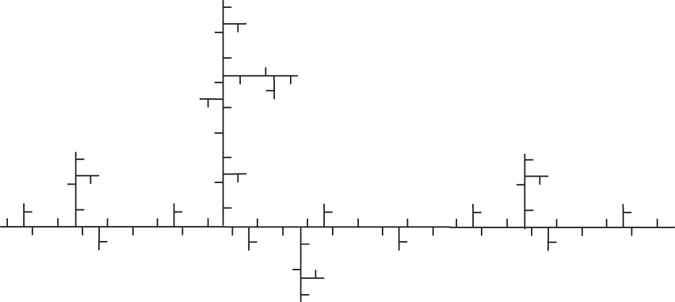
Step 4 of self-similar fractal of model 2.

Taking 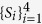, we give a self-similar fractal of model 2 (Figs 17 and 18).


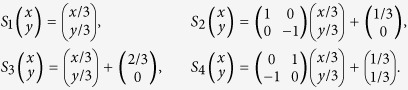


Then the OSC also holds. Let *E*_1_, *E*_2_ be the self-tree of models 1 and 2 respectively. Then


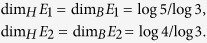


### Moran fractal and random fractal

Fix an infinite sequence 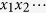 of 1 and 2, we can generate a Moran fractal with mixed model. If *x*_*t*_ = 1 then we take model 1, else we take model 2. Let


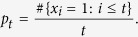


If 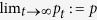 exists, then the corresponding fractal has fractal dimension


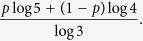


An interesting fact is that this is a deterministic fractal without self-similarity. This is a Moran fractals^31^.

An alternative is a random fractal such that for each time *t*, we can choose model 1 in probability *p* and model 2 in probability 1 − *p*. Then we obtain the above dimension almost surely.

## Ahlfors regularity of networks

### Proof of Theorem 2

By the definition of Ahlfors regularity, we have *d*_*f*_ = *s*.

Suppose 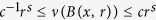. Since the network is covered by *B*_*l*_ balls of radius *l*, that means


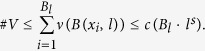


On the other hand, we have *P*_*l*/2_ packing balls of radius *l*/2, which implies


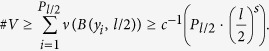


That means


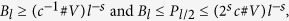


here we use the inequality *B*_2*δ*_ ≤ *P*_*δ*_ ≤ *B*_*δ*/2_ in Lemma 1. Therefore,


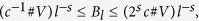


which implies 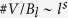, i.e., *d*_*ball*_ = *d*_*B*_ = *s*.

### Proof of Theorem 3

Let *A* = (0, 0) and *B* = (1, 0). Let 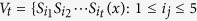 and 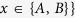.

**Remark 3**. *One node may have distinct codings* 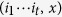 *and* 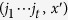 *if* 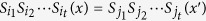. *We also notice that each node has three codings at most*.

Two different nodes *x*, 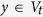 are neighbors if and only if there exists a word 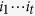 such that


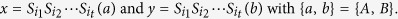


Let d_*t*_ be the geodesic distance on *G*_*t*_.

We denote 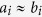 if there is a constant *d* > 0 independent of the index *i* such that *d*^−1^*b*_*i*_ ≤ *a*_*i*_ ≤ *db*_*i*_.

Now, we will prove the following important

**Lemma 2**. *There is a constant c* > 0 *independent of t such that*


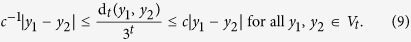


*Proof*. Suppose


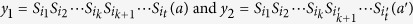


where 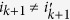. Notice that


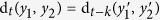


where 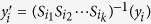. Without loss of generality, we assume that 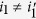.

**Case 1**. If 
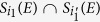
 is empty, then 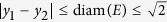 and 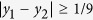, and


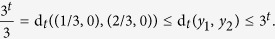


Then (9) follows in this case.□

**Case 2**. If 
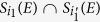
 is non-empty, we may assume that 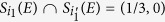 without loss of generality.

For *D* = (1/3, 0), let 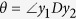. Then there exists 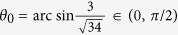 (Fig. 16) such that *θ* ≥ *θ*_0_ (>0). Now,


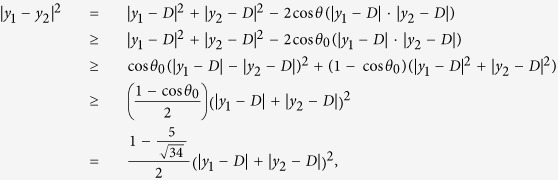


which implies


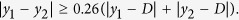


We also have 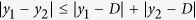. Therefore, we have


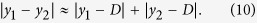


On the other hand,


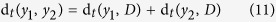


by the tree structure. It follows from (10) and (11) that we only need to verify (9) for the pairs (*y*_1_, *D*) and (*D, y*_2_). By the self-similarity, now we only need to prove the case when 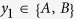.

Without loss of generality, let *y*_1_ = *A* and 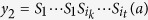 where 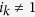. Then


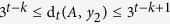


and


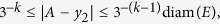


then (9) follows.

Since the OSC holds, then the self-similar measure *μ* with respect to the vector (1/5, 1/5, 1/5, 1/5, 1/5) is Ahlfors *s*-regular for *s* = log 5/log 3.

It follows from the above lemma and Remark 3 that


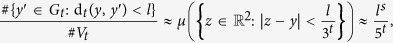


where #*V*_*t*_ = 5^*t*^ + 1. Therefore, we have


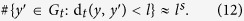


Notice that the constant in (12) is independent of *t*. Now, the growing networks {*G*_*t*_}_*t*_ are Ahlfors *s*-regular.

## Conclusion

We focus on the NP-completeness of minimum ball-covering problem, propose some heuristic ball-covering algorithms such as GOBC, GDBC, VOBC and VGBC, and compare these algorithms with usual RBC algorithm. Inspired by the notion of measure on fractal, a natural measure on the finite graph is obtained such that the measure of every subset is the cardinality of subset. Based on this measure, we revisit the volume dimension *d*_*f*_ and propose the random ball-volume algorithm, which has performance better than the above five minimum covering algorithms due to the NP-completeness. Applying the notion of Ahlfors regularity from fractal geometry, we prove that *d*_*B*_ = *d*_*ball*_ = *d*_*f*_ = *s* if the network is Ahlfors *s*-regular. Finally, we investigate the Ahlfors regularity of a class of self-similar trees and random trees which come from the self-similar fractals and Moran fractals respectively. Although we only prove Theorem 3 for self-similar tree of model 1, but our approach can be applied to many self-similar trees, Moran tree and random trees. Essentially, our approach is to embed our networks into a self-similar (or Moran) fractal (on Euclidean space) satisfying the open set condition, using the Ahlfors regularity of corresponding self-similar (or Moran) measure, we can estimate the volume of balls in networks.

## Additional Information

**How to cite this article**: Wang, L. *et al*. On the Fractality of Complex Networks: Covering Problem, Algorithms and Ahlfors Regularity. *Sci. Rep.* **7**, 41385; doi: 10.1038/srep41385 (2017).

**Publisher's note:** Springer Nature remains neutral with regard to jurisdictional claims in published maps and institutional affiliations.

## Figures and Tables

**Table 1:** *d*_*ball*_ w.r.t. 5 algorithms for the WWW network.

Algorithm	RBC	DGBC	DOBC	VGBC	VOBC
*d*_*ball*_	4.1811	4.5693	5.0805	5.0950	4.2680
